# Influence of energy loss function to the Monte Carlo simulated electron backscattering coefficient

**DOI:** 10.1038/s41598-022-20466-3

**Published:** 2022-10-28

**Authors:** Haotian Chen, Yanbo Zou, Shifeng Mao, M. S. S. Khan, Károly Tőkési, Z. J. Ding

**Affiliations:** 1 Department of Physics, University of Science and Technology of China, Hefei, 230026 Anhui People’s Republic of China; 2 Hefei National Research Center for Physical Sciences at the Microscale, University of Science and Technology of China, Hefei, 230026 Anhui People’s Republic of China; 3 School of Physics & Electronic Engineering, Xinjiang Normal University, Urumchi, 830054 Xinjiang People’s Republic of China; 4 Department of Nuclear Science and Engineering, University of Science and Technology of China, Hefei, 230026 Anhui People’s Republic of China; 5 Institute for Nuclear Research (ATOMKI), Debrecen, EU Hungary

**Keywords:** Theory and computation, Surfaces, interfaces and thin films

## Abstract

We report an improved calculation of the electron backscattering coefficients (BSCs) for beryllium, molybdenum and tungsten at electron energies of 0.1–100 keV based on an up-to-date Monte Carlo simulation method with different input of energy loss function (ELF) data. The electron inelastic cross-section is derived from the relativistic dielectric functional formalism, where the full Penn’s algorithm is applied for the extension of the ELF from the optical limit of $q \to 0$ into the $\left( {q,\omega } \right)$-plane. We have found that the accuracy of energy loss function may affect largely the calculated BSC. We also show that this has close relationship with the *f*- and *ps*-sum rules.

## Introduction

When an electron beam is direct against a thick material, many incident electrons stop moving in the target, but some of them can be scattered backward and escape from the material. These electrons, with their energies higher than 50 eV, are defined as backscattered electrons; while the lower energy electrons are the excited secondary electrons^1^. The properties of these electrons are applied in various fields^2–5^, especially in the scanning electron microscopy (SEM). For the characteristics of these electrons in SEM, signals from secondary electrons are more surface sensitive^6^, making it easy to produce high resolution image of surface topography; backscattered electrons are often used to analyze bulk composition because the signal intensity is highly related to the atomic number of the material. Secondary electron yield, which is defined as the number of secondary electrons per primary electron, is related to bulk electronic properties of the material as well as the surface properties^7–9^; while backscattering coefficient (BSC), which is defined as the number of backscattered electrons per primary electron, is mainly related to the atomic number of the material elements. The study of secondary electron yields and backscattering coefficients for ideal planar surfaces of pure elemental materials has been an important topic since 1960’s for the understanding of SEM image contrast.

Theoretically, Monte Carlo method is an ideal tool for the study of electron-solid interaction, which has been widely used in the fields of electron spectroscopy and electron microscopy. In the past few decades many works have been done and some Monte Carlo models were proposed by using different methods in electron scattering theories^10–23^. In this regard we have developed systematically Monte Carlo simulation models and methods and corresponding codes for various applications to electron beam techniques^24^, e.g. the CTMC-SEM for the simulation of secondary electrons and backscattered electrons emitted from bulk solids as signals in SEM and background in Auger electron spectroscopy, the CTMC-3DSEM for the simulation for complex 3D sample geometries particularly for critical dimension scanning electron microscopic imaging, the CTMC-SES for the simulation of Auger electron and/or X-ray photoelectron signals in surface electron spectroscopies, CTMC-REELS for the simulation of electron elastic peak spectroscopy and reflection electron energy loss spectroscopic spectrum from surfaces, the CTMC-RMC for deriving optical constants of solids from reflection electron energy loss spectroscopy spectra, the CTMC-CHARG for the simulation of specimen charging phenomena in insulators and semiconductors, and CTMC-ATOMIC for the simulation for atomic thin layers with or without substrate particularly for deriving electron inelastic scattering mean free path.

For the importance to nuclear fusion study, the BSCs for beryllium (Be)^24^, molybdenum (Mo) and tungsten (W)^25^ as plasma facing materials in a fusion reactor have been calculated by using the up-to-date Monte Carlo simulation model, CTMC-SEM, based on the Mott’s elastic cross-section^26^ and dielectric functional formalism for the calculation of the inelastic cross-section^27,28^. In those calculations mainly the influences of different elastic cross-sections via the use of different scattering potentials were investigated. We have illustrated that the different elastic cross-sections cannot account for the difference between the measurement data and simulation results of backscattering coefficient for these elements, while the additional CTMC-ATOMIC simulation for the carbon contaminated surface can explain very well the discrepancy: the carbon contamination on the surfaces in a very small amount of several atomic layers can enhance the BSC for the lighter element (Be) but reduce for the heavier elements (Mo and W). Considering the fact that the experimental measurement of completely clean surfaces has been rarely done by simultaneous monitoring the remaining amount of contamination ingredients, mainly carbon and oxygen, the highly reliable experimental data of secondary electron yield and BSC are still missing. Compared with secondary electrons which are quite sensitive to many physical and chemical properties of surfaces due to their low energy character (about several eVs), the backscattered electrons having much higher energies do not suffer the effect of change of work function by a surface treatment procedure. Therefore, it is very promising to build such a reliable theoretical database of electron BSC through the use of Monte Carlo simulation method once a highly accurate physical model is established.

Our previous studies have indicated that the CTMC-SEM model possesses such ability for the accurate simulation of secondary electrons and backscattered electrons for metallic materials. However, with this model some inputs to the calculation of scattering cross sections still have uncertainty. For example, the scattering potential for the calculation of elastic scattering cross section is quite uncertain, which may lead to slight uncertainty of the calculated BSC^24,25^. Furthermore, for the calculation of electron inelastic scattering cross section in the dielectric functional formalism the optical energy loss function (ELF) is a key physical quantity. ELF is defined as the imaginary part of a negative inverse of complex dielectric function $\varepsilon \left( {q,\omega } \right)$, i.e. ${\text{Im}} \left\{ {{{ - 1} \mathord{\left/ {\vphantom {{ - 1} {\varepsilon \left( {q,\omega } \right)}}} \right. \kern-\nulldelimiterspace} {\varepsilon \left( {q,\omega } \right)}}} \right\}$, which describes the electronic excitation of valence and inner-shell electrons in a solid that is responsible for electron energy loss process with the energy transfer $\hbar \omega$ and momentum transfer $\hbar q$. Because of the complexity of electronic excitation in a realistic material the ELF can hardly be accurately obtained at present by a theoretical calculation, mostly the experimentally measured data in the optical limit of $q \to 0$ have been employed in most of the Monte Carlo simulations as well as in the calculations of electron inelastic mean free path (IMFP)^29–31^.

Regards to ELF, one must note that the compiled experimental database^32^ is made of many datasets measured by different researchers in the same or the distinct photon energy regions. These data may differ somewhat, which will affect the calculated inelastic cross sections and, hence, the BSCs. So far the variance of BSC with the uncertainty of input ELF data has not been investigated yet. Similar to the previous work done on the sensitivity of SEM simulation to the model parameters^33^, the aim of the present work is, therefore, to study how the ELF can influence the calculated BSC. For this purpose, we have calculated the inelastic cross sections with different ELF input data, based on the Palik’s database measured by optical methods^32^ and also combined with the data extracted from experimental reflection electron energy-loss spectroscopy (REELS) spectra^34^. We present the BSCs of Be, Mo and W with different ELFs calculated in the incident energy range between 0.1 keV and 100 keV. This calculation leads to improved theoretical data of electron BSCs.

## Monte Carlo model

The up-to-date Monte Carlo simulation model with the latest electron elastic and inelastic scattering cross sections are used in our present calculation. Details of this procedure are described elsewhere^24,25^; here we present only an outline of our calculation procedure.

### Electron elastic scattering

The Mott’s differential cross section^26^ is used to calculate our elastic scattering of electrons:

1 $$\frac{{d\sigma_{e} }}{d\Omega } = \left| {f\left( \theta \right)} \right|^{2} + \left| {g\left( \theta \right)} \right|^{2}$$

where $f\left( \theta \right)$ and $g\left( \theta \right)$ are the scattering amplitudes and can be calculated with the partial wave expansion method,

2 $$f\left( \theta \right) = \frac{1}{2ik}\mathop \sum \limits_{l = 0}^{\infty } \left\{ {\left( {\ell + 1} \right)\left( {e^{{2i\delta_{\ell }^{ + } }} - 1} \right) + \ell \left( {e^{{2i\delta_{\ell }^{ - } }} - 1} \right)} \right\}P_{\ell } \left( {\cos \theta } \right)$$

3 $$g\left( \theta \right) = \frac{1}{2ik}\mathop \sum \limits_{\ell = 1}^{\infty } \left\{ { - e^{{2i\delta_{\ell }^{ + } }} + e^{{2i\delta_{\ell }^{ - } }} } \right\} P_{\ell }^{1} \left( {\cos \theta } \right)$$

where $\delta_{l}^{ + }$ and $\delta_{l}^{ - }$ show spin up and spin down phase shifts of the $\ell$ th partial wave, respectively; $P_{\ell } \left( {\cos \theta } \right)$ and $P_{\ell }^{1} \left( {\cos \theta } \right)$ are the Legendre and the first order associated Legendre functions, respectively.

In this work, the scattering potential contains three parts, i.e., the electrostatic potential, the exchange potential and the correlation-polarization potential. The Fermi distribution and the Dirac–Fock electron density^35^ are used to determine the nuclear and electronic charge-density, respectively. In addition, the Furness-McCarthy exchange potential^36^ and the correlation-polarization potential based on the local-density-approximation^37^ are also considered. The Mott’s cross section is calculated with the ELSEPA program^38^.

### Electron inelastic scattering

The dielectric functional formalism is used to determine the inelastic scattering cross sections of electrons. In this model, the differential inverse inelastic mean free path (DIIMFP) for moving electrons in a material is written as:

4 $$\frac{{d^{2} \lambda_{in}^{ - 1} }}{{d\left( {\hbar \omega } \right)dq}} = \frac{{2\gamma^{2} }}{1 + \gamma }\frac{1}{{\pi a_{0} E}}{\text{Im}}\left\{ {\frac{ - 1}{{\varepsilon \left( {q,\omega } \right)}}} \right\}\frac{1}{q}$$

where $\gamma = 1 + {E \mathord{\left/ {\vphantom {E {\left( {m_{0} c^{2} } \right)}}} \right. \kern-\nulldelimiterspace} {\left( {m_{0} c^{2} } \right)}}$ is the relativistic correction factor, $a_{0}$ is the Bohr radius and $\lambda_{in}^{{}}$ is the electron inelastic mean free path (IMFP). $\varepsilon \left( {q,\omega } \right)$ is the complex dielectric function of a medium. The probability of the inelastic scattering events is determined by the ELF. Penn has suggested an algorithm, known as the full Penn algorithm (FPA)^27^, for the extension of the optical ELF, ${\text{Im}} \left\{ {{{ - 1} \mathord{\left/ {\vphantom {{ - 1} {\varepsilon \left( {0,\omega } \right)}}} \right. \kern-\nulldelimiterspace} {\varepsilon \left( {0,\omega } \right)}}} \right\}$, from the optical limit of $q \to 0$ into the $\left( {q,\omega } \right)$-plane. Using the Lindhard dielectric function $\varepsilon_{L} \left( {q,\omega ;\omega_{p} } \right)$, the ELF is written as:

5 $${\text{Im}}\left\{ {\frac{ - 1}{{\varepsilon \left( {q,\omega } \right)}}} \right\} = \int_{0}^{\infty } {g\left( {\omega_{p} } \right){\text{Im}}\left\{ {\frac{ - 1}{{\varepsilon_{L} \left( {q,\omega ;\omega_{p} } \right)}}} \right\}d\omega_{p} }$$

where $g\left( \omega \right)$ is the expansion coefficient, which is related to the optical ELF by,

6 $$g\left( \omega \right) = \frac{2}{\pi \omega }{\text{Im}}\left\{ {\frac{ - 1}{{\varepsilon \left( {0,\omega } \right)}}} \right\}$$

To check the accuracy of the ELFs used, one can apply the oscillator strength sum rule (*f*-sum rule) and the perfect screening sum rule (*ps*-sum rule)^39^. The *f*-sum rule $Z_{eff}$ is given by,

7 $$Z_{eff} = \frac{2}{{\pi \Omega_{P}^{2} }}\int\limits_{0}^{{\omega_{\max } }} {\omega {\text{Im}} } \left\{ {\frac{ - 1}{{\varepsilon \left( \omega \right)}}} \right\}d\omega$$

where $\hbar \Omega_{P} = \sqrt {{{4\pi n_{a} e^{2} } \mathord{\left/ {\vphantom {{4\pi n_{a} e^{2} } m}} \right. \kern-\nulldelimiterspace} m}}$. The expectation value of $Z_{eff}$ must be the atomic number $Z$ of the atom, or the total number of electrons per atom or molecule, when $\omega_{\max } \to \infty$. The *ps*-sum rule $P_{eff}$ can be obtained from the Kramers–Kronig relation as^40,41^:

8 $$P_{eff} = \frac{2}{\pi }\int\limits_{0}^{{\omega_{\max } }} {\frac{1}{\omega }{\text{Im}} } \;\left\{ {\frac{ - 1}{{\varepsilon \left( \omega \right)}}} \right\}d\omega + {\text{Re}} \left\{ {\frac{1}{\varepsilon \left( 0 \right)}} \right\}$$

where ${\text{Re}} \left\{ {{1 \mathord{\left/ {\vphantom {1 {\varepsilon \left( 0 \right)}}} \right. \kern-\nulldelimiterspace} {\varepsilon \left( 0 \right)}}} \right\} = 0$ for conductors. The expectation value of $P_{eff}$ is thus unity when $\omega_{\max } \to \infty$.

### Monte Carlo simulation

When an electron is incident into a sample, it will suffer elastic and inelastic collisions and change the direction of movement and kinetic energy, respectively. The scattering angle and the energy loss can be sampled by the respective differential cross section with random numbers in a Monte Carlo simulation. The high energy secondary electrons (> 50 eV) are included in the calculation of BSC. If the lost energy $\hbar \omega$ is less than the binding energy of the corresponding inner-shell, $\hbar \omega < E_{B}$, where $E_{B}$ is the smallest binding energy of the observable ionization edge of an inner-shell presented in the optical ELF, then a secondary electron is assumed to be excited from Fermi sea by transferring $\hbar \omega$ energy from primary electron to a valence electron of energy with the excitation probability being proportional to a joint density of states of free electrons. If $\hbar \omega > E_{B}$, the secondary electron is excited from the inner-shell and has kinetic energy of $\hbar \omega - E_{B}$. In addition, the relaxation of excited atoms may proceed via the emission of an Auger electron or a photon. However, the contribution of Auger electrons to the BSC is negligible due to the low probability of the inner-shell ionization.

After undergoing multiple elastic and inelastic scatterings inside the sample, some electrons can reach back to the surface. These electrons can then escape from the sample with a certain probability, i.e. the transmission function $T$. In this work, a quantum mechanical transmission function is used^8^,

9 $$T\left( {E,\beta } \right) = \left\{ {\begin{array}{*{20}l} {\frac{{4\sqrt {1 - {{U_{0} } \mathord{\left/ {\vphantom {{U_{0} } {E\cos^{2} \beta }}} \right. \kern-\nulldelimiterspace} {E\cos^{2} \beta }}} }}{{\left[ {1 + \sqrt {1 - {{U_{0} } \mathord{\left/ {\vphantom {{U_{0} } {E\cos^{2} \beta }}} \right. \kern-\nulldelimiterspace} {E\cos^{2} \beta }}} } \right]^{2} }},} \hfill & {{\text{if }}\;E\cos^{2} \beta > U_{0} ;} \hfill \\ {0,} \hfill & {{\text{otherwise}}{.}} \hfill \\ \end{array} } \right.$$

where $\beta$ is the angle between the electron moving direction and the surface normal, and $U_{0}$ is the surface barrier which is the sum of work function and Fermi energy^17^. According to the kinetic energy, an escaped electron is counted either as a true secondary electron (< 50 eV) or a backscattered electron (> 50 eV). The BSC is defined by the number of backscattered electrons per an incident electron while the secondary electron yield is similarly defined by the number of secondary electrons per an incident electron.

### Energy loss function data

**Figure 1:**
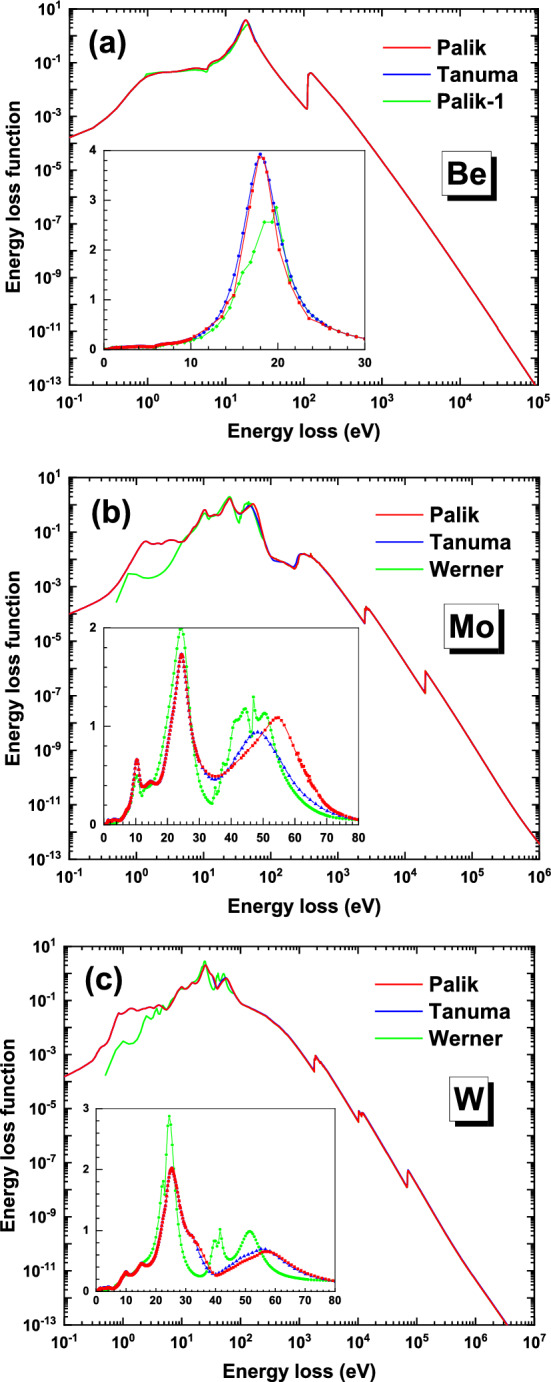
The energy loss functions of (**a**) beryllium, (**b**) molybdenum and (**c**) tungsten, where the data properties are described in Tables 1 and 2.

In Palik’s database there are several datasets of optical constants^42–44^; then we have combined them to form three optical ELFs, named as “Tanuma”, “Palik” and “Palik-1”, listed in Table 1, which are used as the input to the Monte Carlo calculation. The “Tanuma”-ELF was actually used in the calculation of IMFP^45^, where the data source has not been mentioned. These optical ELFs of beryllium are shown in Fig. 1a. The data in the photon energy $\hbar \omega$ (or electron energy loss) range of 0.02–300 eV are all taken from Palik’s database^32^ but grouped with different sources, and in the range of 300 Ev–30 keV and 30 keV–10 MeV from Henke et al.^46^ and EPDL97 database^47^, respectively, whose numerical data can be found elsewhere^48,49^. The other two different ELFs are named as “Palik” and “Palik-1” to distinguish them for which they are very different in the peak region of ELF.

**Table. 1:** The optical ELF data used for Be.

Name	Photon energy range and the corresponding datasets				
Palik	0.02–10 eV Arakawa^42^	10.38–25.95 eV Toots^43^	26–300 eV Arakawa^42^	300 eV-30 keV Henke^46^	30 keV-10 MeV EPDL97^47^
Tanuma	0.02–300 eV Arakawa^42^, in: Tanuma^45^				
Palik-1	0.02–0.9 eV Arakawa^42^	0.96–21.38 eV Seignac^44^	21.5–300 eV Arakawa^42^

The ELFs of Mo and W are shown in Fig. 1b and Fig. 1c, respectively. The data are taken from Palik’s database^32^, the REELS spectra^34^, the Henke et al.^46,48^ and EPDL97 database^47,49^ in the different photon energy ranges. The three combined ELFs are named as “Palik”, “Tanuma” and “Werner”, whose data in the low energy range are taken from either Palik’s or Werner’s database. The data combination of these ELFs are described in Table 2. Note that even though Tanuma et al.^45^ have described that their data property is the same as that of “Palik”, but actually in the range of 40–80 eV their data are somewhat different from the original data in the Palik’s database. Perhaps certain interpolation was also made in this energy range and, therefore, we name their ELF data as “Tanuma”.

**Table. 2:** The optical ELF data used for Mo and W.

Element	Name	Photon energy range and the corresponding datasets			
Mo	Palik	0.1–30 eV Juenker^50^ and Weaver^51^, in: Palik^32^	31–39 eV Interpolation^45^	40 eV–30 keV Henke^48^	30 keV–10 MeV EPDL97^49^
	Tanuma			40 eV-30 keV Henke^46^, in: Tanuma^45^	
	Werner	0.5–70.5 eV Werner^34^	71–80 eV Interpolation	80 eV–30 keV Henke^48^	
W	Palik	0.05–33.5 eV Weaver^52^	34–39 eV Interpolation^45^	40 eV–30 keV Henke^48^	
	Tanuma			40 eV–30 keV Henke^46^, in: Tanuma^45^	
	Werner	0.5–70.5 eV Werner^34^	71–80 eV Interpolation	80 eV–30 keV Henke^48^	

**Figure 2:**
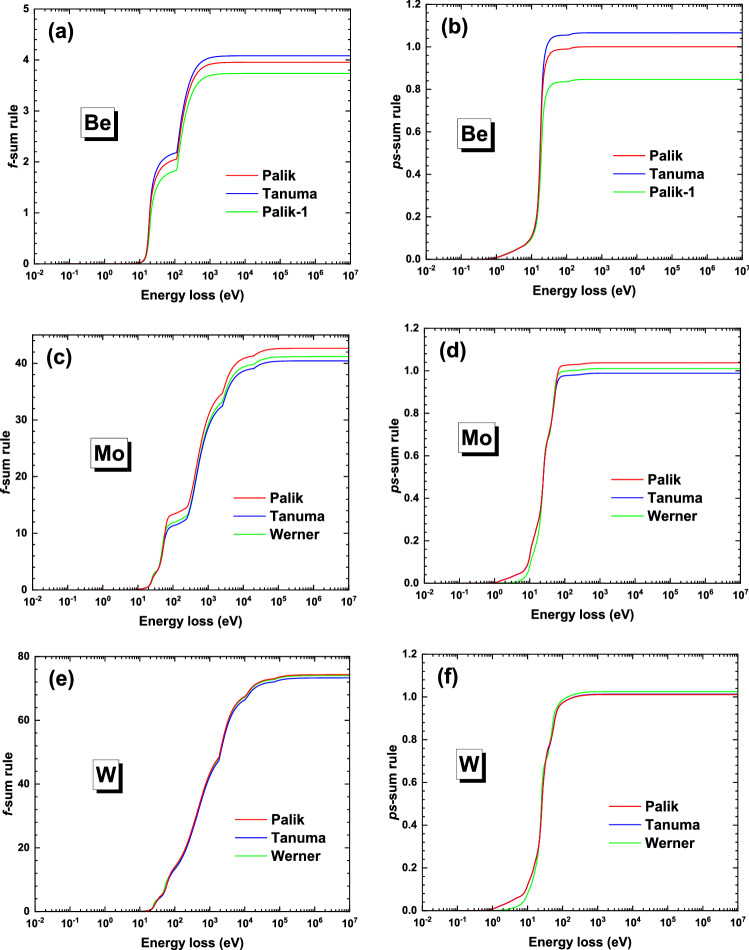
The *f*- and *ps*-sum rules for (**a**) (**b**) beryllium, (**c**) (**d**) molybdenum and (**e**) (**f**) tungsten, respectively.

Figure 2 shows the $Z_{eff} \left( {\omega_{\max } } \right)$ and $P_{eff} \left( {\omega_{\max } } \right)$ in Eqs. 7 and 8 as functions of integration upper limit $\omega_{\max }$ for these ELFs of the three elements. Table 3 presents the corresponding *f*-sum and the *ps*-sum rules when $\omega_{\max } \to \infty$. Note that the sum rule values are quite different among the different data groups (i.e. “Palik”, “Tanuma” & “Palik-1” for Be, and “Palik”, “Tanuma” & “Werner” for Mo and W), while the present estimation of sum rules for “Tanuma”-ELFs is slightly different from their reported values^45^. Comparatively, “Palik”-ELF data is the best for Be because the relative errors for both the *f*- and the *ps*-sum rules are the smallest. However, for Mo and W it is hard to judge which one among “Palik”-, “Tanuma”- and “Werner”-ELFs is the best because while one of the errors for *f*- and the *ps*-sum rules is smaller another one would be greater for a specific ELF. This will affect the judgement on the quality of the computed BSC. The *ps*-sum rule is dominated by the accuracy of the low energy part of ELF, as can be understood by the $\omega^{ - 1}$-factor in the integration of Eq. (8), which is the most important energy loss region in electron inelastic scattering responsible for the valence electron excitation; while the accuracy of *f*-sum rule is dominated by the accuracy of the high energy part of ELF by the $\omega$-factor in the integration of Eq. (7). Therefore, different sum rules emphasize the different energy regions being low or high; but, this is just a simple overall estimation and the accuracy of an ELF in a short photon energy range cannot be given definitely.

**Table. 3:** The *f*- and *ps*-sum rules and their relative errors for Be, Mo and W, where the data in parentheses were from Tanuma et al. ^45^.

		*f*-sum rule		*ps*-sum rule	
		Value	Relative error (%)	Value	Relative error (%)
Be	Palik	3.95	− 1.15	1.00	0.04
	Tanuma	4.08 (4.09)	2.05 (2.4)	1.07 (1.066)	6.62 (6.6)
	Palik-1	3.73	− 6.63	0.85	− 15.31
Mo	Palik	42.66	1.58	1.04	3.78
	Tanuma	40.43 (39.53)	− 3.73 (− 5.9)	0.98 (0.989)	− 1.11 (− 1.1)
	Werner	41.18	− 1.96	1.01	1.05
W	Palik	74.32	0.43	1.01	1.13
	Tanuma	73.31 (71.39)	− 0.93 (− 1.3)	1.01 (1.013)	1.30 (1.3)
	Werner	74.05	0.07	1.02	2.49

## Results and discussions

**Figure 3:**
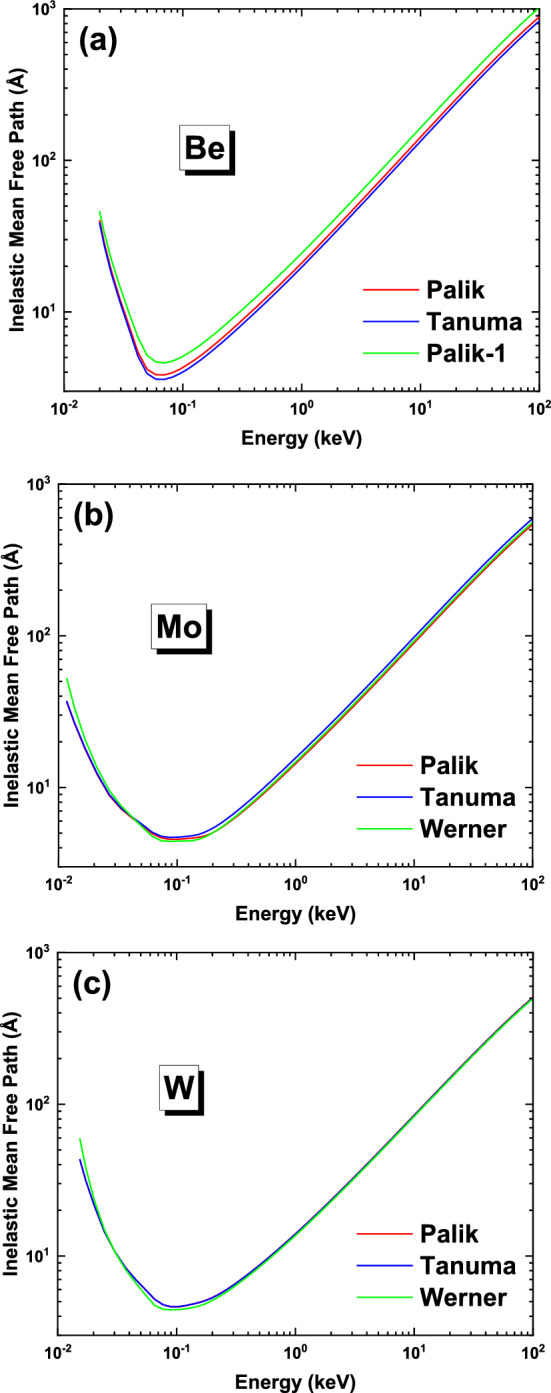
The inelastic mean free path for (**a**) Be, (**b**) Mo and (**c**) W, which are calculated from the ELFs in Fig. 1.

From an ELF we have at first obtained the energy dependent IMFP, $\lambda_{in}^{{}} \left( E \right)$, which is the average distance that an electron will travel in the material before losing energy and is also proportional to the inverse of inelastic scattering cross section, by double integration of DIIMFP in Eq. (4) where the upper limit for integration over the energy loss $\hbar \omega$ is $E - E_{F}$, where $E$ is electron kinetic energy and $E_{F}$ is the Fermi energy. Higher IMFP value means that an electron of kinetic energy *E* can travel a longer distance before losing an energy, implying that it is easier to be backscattered from the material. The behavior of IMFP on kinetic energy $E$ can then be used to explain the dependence of BSC, $\eta \left( {E_{p} } \right)$, on primary energy $E_{p}$ later. From Fig. 3a it can be seen that for beryllium the three IMFP curves have no intersection; the “Tanuma”-IMFP curve is always lower than “Palik”-IMFP curve from the low energy region to the high energy region, and “Palik”-IMFP curve is always lower than “Palik-1”-IMFP curve. This behavior is consistent with the sum rule results shown in Table 3, where the positive sum rule errors for “Tanuma”-ELF indicate that this ELF is overestimated in the whole range of photon energy $\hbar \omega$ and, hence, the resultant IMFP is underestimated in the whole range of kinetic energy *E*. The very small sum rule errors for “Palik”-ELF represent the fact that the obtained “Palik”-IMFP should be the most reasonable, while the large negative errors for “Palik-1”-ELF result in the much overestimated “Palik-1”-IMFP values.

For Mo and W, such an analysis becomes a little bit complex. In case of Mo, “Palik”- and “Werner”-IMFP curves have two intersection points: one is below 50 eV, and another one is at about 200 eV. The “Palik”-IMFP is larger than “Werner”-IMFP in the range of 50–200 eV, and smaller otherwise. This behavior is related to the different sum rule errors of the “Palik”- and “Werner”-ELFs. The positive and negative errors of *ps*- and *f*-sum rules, respectively, for the “Werner”-ELF means that the ELF is slightly overestimated in the low $\hbar \omega$ range, but underestimated at high photon energies. Correspondingly, the “Werner”-IMFP is slightly underestimated and overestimated at low and high energies, respectively. In comparison, the “Palik”-ELF is overestimated in the low $\hbar \omega$ range, and slightly overestimated at high photon energies. Therefore, the “Palik”-IMFP is more and slightly underestimated at low and high energies, respectively. The analysis indicates that below 50 eV, both IMFPs are underestimated and the simulation in this energy range would result in a large error. Fortunately, for the calculation of BSC the primary energy must be limited to 50 eV above. Below 40 eV of photon energy, “Tanuma”- and “Palik”-ELFs are the same, while in the range of 40–80 eV they are in fact different, as can be seen from Fig. 1b. Therefore, “Tanuma”-IMFP agrees with “Palik”-IMFP at low electron kinetic energies below 40 eV; but at higher kinetic energies, “Tanuma”-IMFP is the largest among the three curves because of its negative and comparatively large *f*-sum rule error, which is expected to result in an overestimated BSC values.

In case of W, the “Palik”- and “Werner”-IMFP curves intersect at about 20 eV above which the “Palik”-IMFP is always greater than the “Werner”-IMFP. One can see from Table 3 that, both the sum rule errors are positive for both the “Palik”- and “Werner”-ELFs. However, the “Palik”-ELF is more accurate at low photon energy region while the “Werner”-ELF is more accurate at high photon energy region; then above 20 eV the “Werner”-IMFP is underestimated and the “Palik”-IMFP is more accurate. “Tanuma”-ELF differs from “Palik”-ELF very slightly in the photon energy range of 35–80 eV, and the sum rule errors of “Tanuma”-ELF is larger but the *f*-sum rule is negative. This implies that the “Tanuma”-IMFP is a little bit overestimated at high kinetic energies and, hence, the BSC for the “Tanuma” curve. However, it can be seen from Fig. 3c that the difference between “Tanuma”- and “Palik”-IMFPs is still negligible.

**Figure 4:**
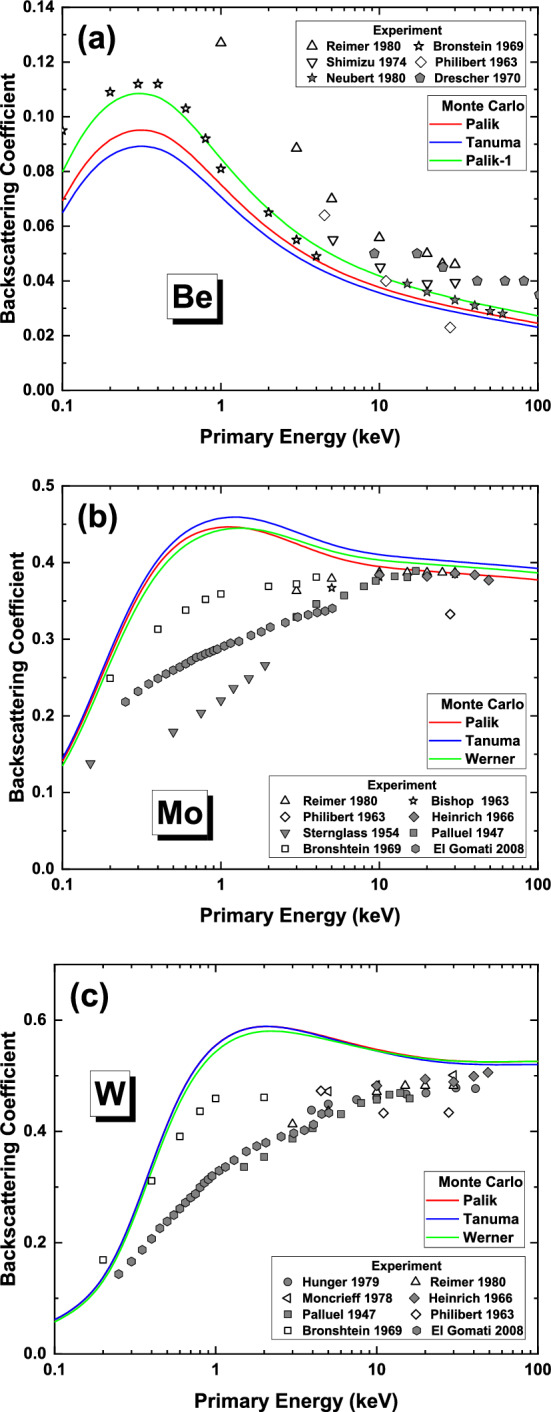
Calculated BSCs of (**a**) beryllium, (**b**) molybdenum and (**c**) tungsten. The Monte Carlo simulation results are compared with experimental data^53–65^.

We have performed the Monte Carlo simulation for the energy spectra of backscattered electrons for primary electrons vertically incident on the ideal smooth and clean surfaces of Be, Mo and W. The calculated range of primary electron energy $E_{p}$ is from 0.1 keV to 100 keV for each material. For each primary energy, 1 × 10^8^ primary electron trajectories were traced. The integration of energy spectra over kinetic energy $E$ gives directly the BSC, $\eta \,\left( {E_{p} } \right)$; therefore, for each $E_{p}$ actually the BSC is contributed by varied electron energies corresponding to the IMFP, $\lambda_{in}^{{}} \,\left( E \right)$, shown in Fig. 3. At low primary energy, only the low energy electrons having low energy loss in ELF can play a role and *ps*-sum rule is more effective; at high primary electron, not only the low energy electrons but also high energy electrons contribute to BSC and, therefore, *f*-sum rule becomes more important. Figure 4 shows the simulated BSCs with different ELFs for the three elemental solids.

For Be the BSCs obtained with three different ELFs in Fig. 4a show the same trend of variation with sum rule errors as IMFP in Fig. 3a. Clearly, larger IMFPs result in greater BSCs because of less electron inelastic scattering would happen during electron transport. In our previous work^24^, the calculations were performed with “Tanuma”-ELF. Because of the positive errors of sum rules, the inelastic scattering cross section is overestimated; hence, “Tanuma”-IMFP and “Tanuma”-BSC are underestimated. It seems from Fig. 4a that the “Palik-1”-BSC is the best when compared with the Bronstein experimental data^53^, but this is not true because the “Palik-1”-ELF is very inaccurate, as indicated by the sum rules. As we have shown in Table 3 that “Palik”-ELF is the best and, hence, “Palik”-BSC should be the most reasonable although it is also slightly overestimated at high energies. The previous study has demonstrated the importance of carbon contamination in the explanation of experimental data^24^. Considering the carbon contamination of one monolayer, the present “Palik”-BSC would agree more excellently with the Bronstein experimental data^53^ than the previous calculation^24^ done with “Tanuma”-ELF when contamination is considered. This fact indicates that the sum rule analysis is indeed useful in the judgement of data quality for a Monte Carlo simulation of BSC if the optical ELF is employed for modeling electron inelastic scattering. The use of more accurate “Palik”-ELF thus improves the theoretical simulation of BSC for Be.

Regarding to Mo and W, the previous work^25^ has employed “Tanuma”-ELF. Using the same analysis as above, we can also infer the quality of the simulated BSCs. For Mo, since the “Tanuma”-ELF is well underestimated while “Palik”-ELF is somewhat overestimated, the corresponding “Tanuma”- and “Palik”-BSCs would be overestimated and underestimated, respectively. Observation of the calculation results in Fig. 4b indeed supports such reasoning. It can be seen that “Palik”-BSC agrees excellently with experimental data at very high energies above 10 keV. Our previous calculation has shown that, in contrast to the light element Be the carbon contamination of solid surfaces of heavy elements, like Mo and W here, would tend to reduce the BSC mostly in the primary energy region of 10^−1^–10^0^ keV. 1–2 monolayers of carbon on Mo surface would present better agreement with Bronstein’s experimental data^53^. “Werner”-BSC should be between “Tanuma”- and “Palik”-BSCs, as suggested by the sum rules. For W, the sum rule values for the three ELFs are comparable and quite small, therefore, one can hardly find remarkable difference between the corresponding three IMFP curves and also BSC values.

## Conclusion

In this work, we have considered the effect of different ELFs on the Monte Carlo simulated electron BSCs for Be, Mo and W in primary energy range of 0.1–100 keV. The detailed analysis of *f*- and *ps*-sum rule results shows that, the *f*-sum rule for ELF emphasizes the calculation accuracy at high primary energy while *ps*-sum rule dominates the accuracy at low primary energy; the large negative/positive errors of *f*- or *ps*-sum rules can lead to overestimation/underestimation of BSC in the region of high- or low-primary energy. Particularly, for Be this effect is quite obvious and the present calculation with “Palik”-ELF is better than the previous work with “Tanuma”-ELF. For Mo, the present “Palik”-ELF also improves the previous calculation; but for W, we have not found remarkable difference with previous results because the sum rule errors are all small. The use of an accurate ELF with smaller errors of *f*- and *ps*-sum rules is quite important for theoretical calculation of BSC towards building a database.

## Data Availability

The datasets generated during and/or analyzed during the current study are available from the corresponding authors on reasonable request.
